# Congenital atlanto-occipital dislocation in a patient with Down syndrome: a case report

**DOI:** 10.1007/s00256-023-04297-5

**Published:** 2023-02-11

**Authors:** Ryoko Onodera, Ryuji Sakamoto, Yuki Taniguchi, Shima Hirai, Yoshitaka Matsubayashi, So Kato, Yasushi Oshima, Sakae Tanaka

**Affiliations:** 1 Department of Orthopedic Surgery, The University of Tokyo Hospital, 7-3-1 Hongo, Bunkyo-Ku, Tokyo, 113-8655 Japan; 2 Surgical Center, The University of Tokyo Hospital, 7-3-1 Hongo, Bunkyo-Ku, Tokyo, 113-8655 Japan

**Keywords:** Atlanto-occipital dislocation, Os odontoideum, Down syndrome, Basilar invagination, Bifurcated internal occipital crest

## Abstract

Down syndrome, also known as trisomy 21, is associated with congenital cervical spine abnormalities, including atlantoaxial instability with or without os odontoideum, atlanto-occipital instability, and hypoplasia of the atlas. Herein, we report a case of Down syndrome complicated by congenital atlanto-occipital dislocation. The patient presented with severe cervical myelopathy at 13 years of age after a 10-year follow-up. Radiography and computed tomography revealed os odontoideum protruding into the foramen magnum and congenital anterior atlanto-occipital dislocation. Additionally, a bifurcated internal occipital crest with a thinned central portion of the occipital bone was noted. Magnetic resonance imaging revealed kyphotic alignment of the spinal cord with severe compression at the foramen magnum level. As the neurological impairment was partially improved by halo vest immobilization, we performed in situ O-C2 fusion with an iliac autograft and decompression of the foramen magnum and posterior arch of C1. An improvement was observed immediately after surgery. Two years after surgery, radiography and computed tomography showed solid O-C2 segment fusion. The accumulation of similar cases is essential for determining the prognosis or optimal treatment for this rare congenital condition.

## Introduction

Down syndrome (OMIM 190685) is caused by trisomy of all or a critical portion of chromosome 21 and can be associated with congenital cervical spine abnormalities, including atlantoaxial instability with or without os odontoideum, atlanto-occipital instability, and hypoplasia of the atlas [1–3]. Herein, we report the first case of Down syndrome complicated by congenital atlanto-occipital dislocation, which demonstrated progressive myelopathy and was successfully treated surgically.

## Case report

### Presentation and examination

**Fig. 1:**
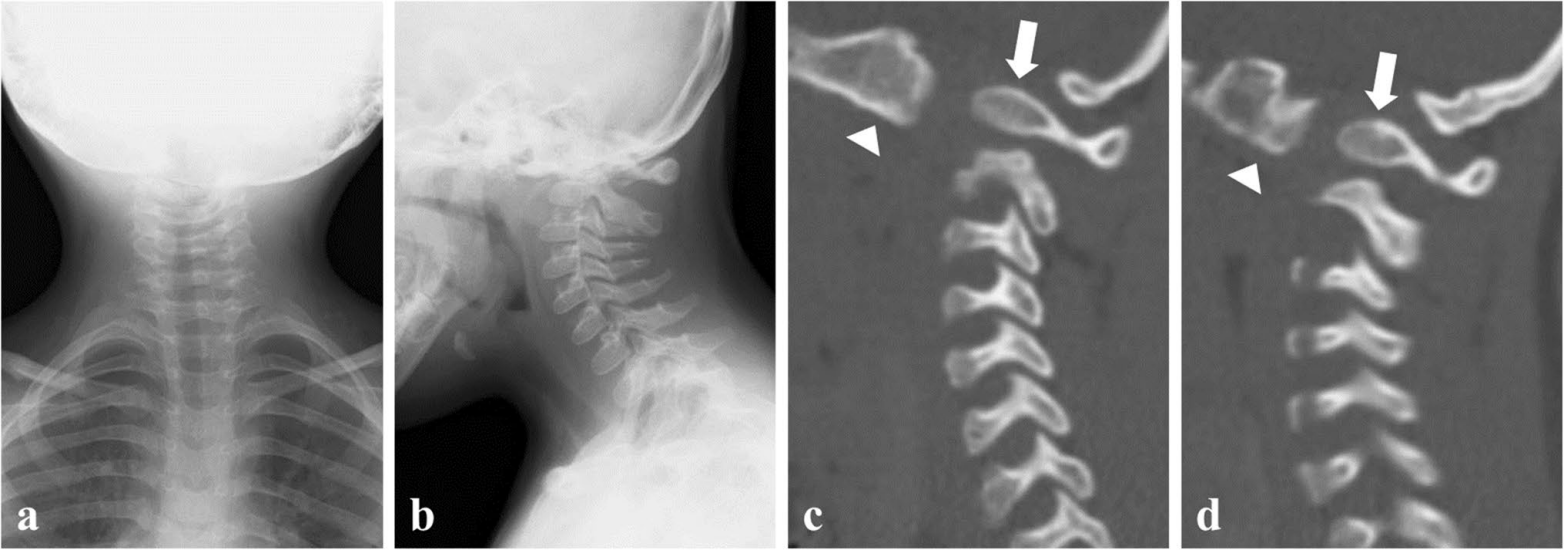
Cervical spine images at 3 years old. **a**, **b** Anterior–posterior and lateral plain radiographs of the cervical spine at 3 years old. **c**, **d** Reconstructed parasagittal computed tomography of the cervical spine at 3 years old revealing anteriorly displaced right (**c**) and left (**d**) occipital condyles (white arrow heads) to the C1 lateral masses (white arrows)

**Fig. 2:**
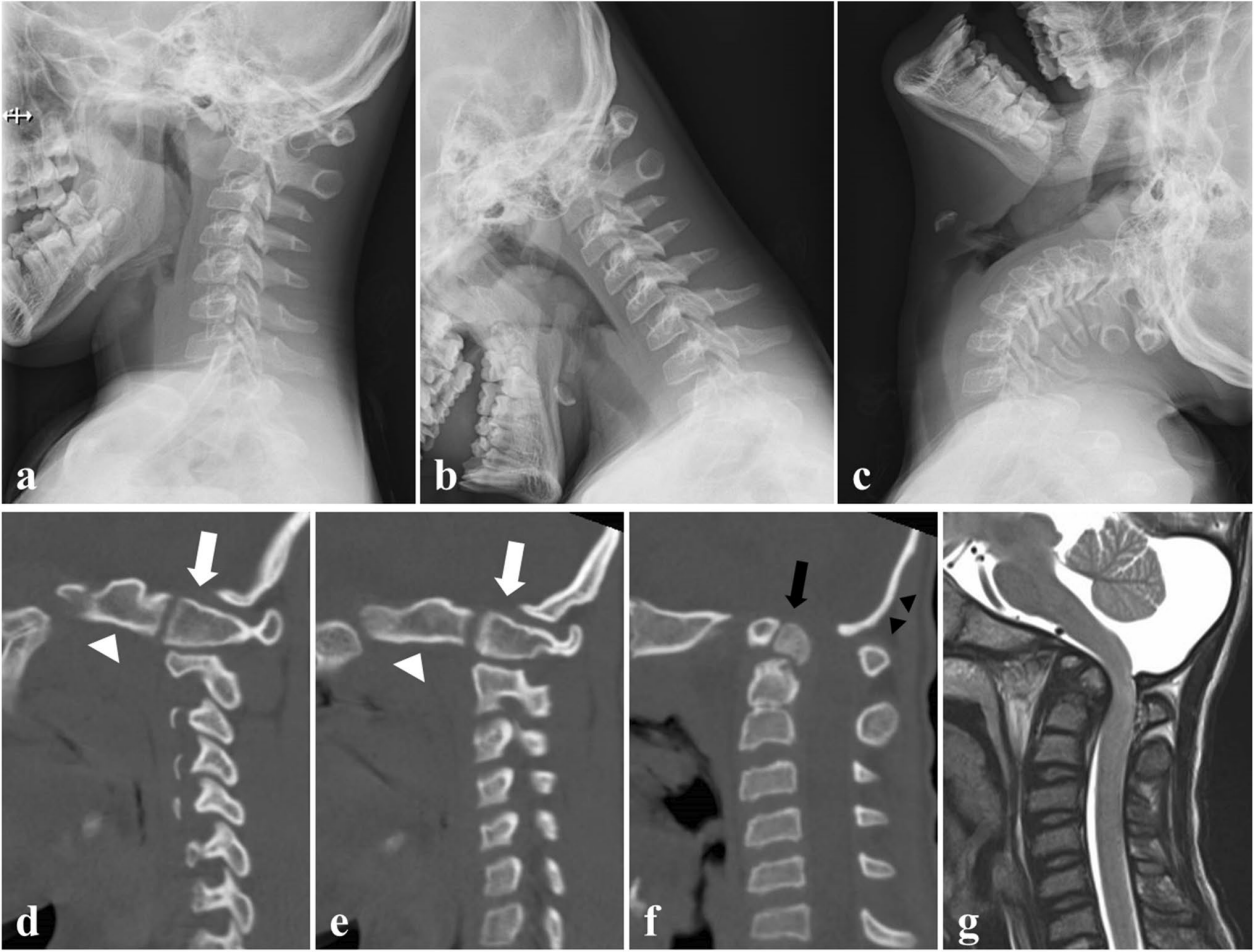
Preoperative cervical spine images at 13 years old. **a**-**c** Dynamic lateral plain radiographs of the cervical spine showing atlantoaxial instability with basilar invagination and O-C2 kyphosis. **d**, **e** Parasagittal reconstruction computed tomography showing vertical O-C1 joints formed by anteriorly displaced bilateral occipital condyles (white arrow heads) and C1 lateral masses (white arrows). **f** Midsagittal reconstruction computed tomography demonstrated os odontoideum (black arrows) protruding into the foramen magnum and O-C2 kyphosis with a thinned central portion of the occipital bone (black arrow heads). **g** T2-weighted sagittal magnetic resonance imaging showing kyphotic alignment of the spinal cord with severe compression at the foramen magnum level

The patient first presented to our department at 3 years of age for abnormal cervical radiography findings. He was diagnosed with Down syndrome at birth. His family history was unremarkable. Radiography showed basilar invagination and kyphotic O-C2 alignment (Fig. 1a, b). Sagittal reconstruction computed tomography demonstrated that the bilateral occipital condyles were anteriorly displaced to the C1 lateral masses, forming vertical O-C1 joints (Fig. 1c, d). He had no history of trauma; therefore, this condition was diagnosed as congenital atlanto-occipital dislocation. We started an annual outpatient clinic follow-up because he showed no signs of myelopathy. At 13 years of age, the patient presented with progressive gait disturbance and difficulty sitting due to cervical myelopathy. Cervical radiography revealed a mobile atlantoaxial segment with basilar invagination and O-C2 kyphosis (Fig. 2a–c). Computed tomography revealed os odontoideum protruding into the foramen magnum with bilateral atlanto-occipital dislocation (Fig. 2d–f). Magnetic resonance imaging showed kyphotic alignment of the spinal cord with severe compression at the foramen magnum level (Fig. 2g).

### Surgery

**Fig. 3:**
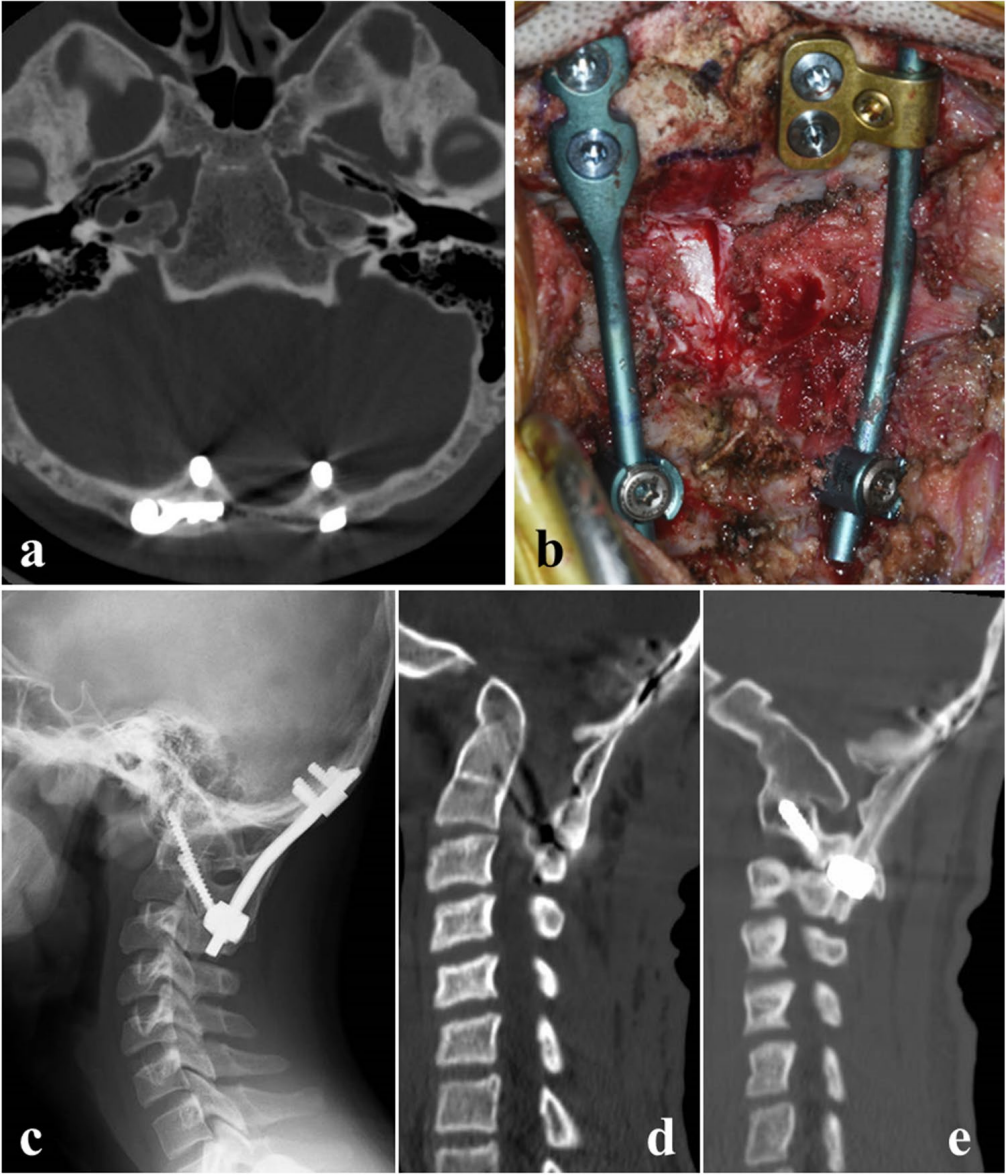
Surgery for upper cervical myelopathy caused by a congenital atlanto-axial dislocation complicated by os odontoidum. **a** Postoperative axial computed tomography demonstrating occipital screws placed bilaterally into the bifurcated internal occipital crests. **b** Intraoperative photograph showing decompression of the foramen magnum and posterior arch of C1 with occipital plates separately placed bilaterally. **c** Lateral radiograph of the cervical spine 2 years after surgery. **d**, **e** Sagittal reconstruction computed tomography 2 years after surgery showing solid fusion of the O-C2 segment. Note that the os odontoideum is fused to the base of the axis

The neurological impairment was partially attributed to upper cervical instability associated with os odontoideum because of partial neurological symptom improvement by the halo vest immobilization. Hence, we performed in situ O-C2 fusion using an iliac autograft and decompression of the foramen magnum and posterior arch of C1, in lieu of reducing the congenital atlanto-occipital dislocation. Due to a bifurcated internal occipital crest with a thinned central portion of the occipital bone, we bilaterally placed the occipital plates separately (Fig. 3a, b). The neurological impairment improved immediately after surgery, and the patient showed no myelopathy 2 years after surgery. Radiography and computed tomography demonstrated solid O-C2 segment fusion, including fusion of the os odontoideum to the base of the axis (Fig. 3c–e).

## Discussion

There are no reports describing congenital atlanto-occipital dislocation; most reported cases have been trauma-related [4–6]. Down syndrome is frequently complicated by congenital craniovertebral anomalies, which include atlanto-occipital instability, atlantoaxial instability, hypoplasia of the atlas, C1 lateral mass morphometric anomaly, basilar invagination with occipitoatlantal assimilation, bifida anterior and/or posterior altantal arches, condylar hypoplasia, ossiculum terminale, and os odontoideum [1–3, 7–10]. However, the etiology of these congenital upper cervical anomalies found in Down syndrome remains unelucidated. Hence, the congenital atlanto-occipital dislocation in this case might have occurred as a part of various congenital cervical malformations in Down syndrome. Despite the high prevalence of these congenital anomalies in patients with Down syndrome, the majority of these cases are asymptomatic, with estimates of symptomatic disease ranging from 1 to 2% [7, 11–13]. Among the previously reported congenital craniovertebral anomalies in Down syndrome, hypoplasia of the atlas and os odontoideum were concomitantly found in the present case. However, the systematic understanding of craniovertebral anomalies in Down syndrome remains a challenge.

The optimal treatment for this congenital atlanto-occipital condition will be the subject of future studies. In this case, preoperative dynamic radiography of the cervical spine revealed limited cephalocaudal motion of the atlanto-occipital joint; therefore, reduction of the atlanto-occipital dislocation was presumed to be technically demanding. Hence, we conducted an in situ fusion and decompression, which was motivated by the partial neurological recovery from external halo vest immobilization alone. Although the short-term clinical course was favorable in our case, long-term follow-up and accumulation of similar cases are essential for determining the optimal treatment for this condition.

Additionally, it remains to be determined whether surgery before the development of myelopathy is recommended in patients with congenital atlanto-occipital dislocation, because the prognosis or natural course of this congenital condition is completely unknown. Retrospectively, our case may have benefitted from prophylactic surgery, given the preoperative severe and rapid neurological deterioration. However, determining the significance of prophylactic surgery for this congenital condition may be premature as neurological impairment in this case may have been affected by concomitant os odontoideum, a well-known predictive factor for neurological deterioration and a proposed indicator for prophylactic surgery due to its high-grade instability [14–17]. Nevertheless, because the natural course of the patients with congenital atlanto-occipital dislocation alone is completely unknown, presently, the optimal timing for surgery in these cases may be at the time of the development of myelopathy. In that respect, non-surgical management before the development of myelopathy in these cases can be another matter of debate. It is generally recognized that patients with biomechanically significant bony anomalies at the occipitoatlantal junction are a special high-risk group and should be studied carefully [18]. Furthermore, a recent consensus study regarding the management of pediatric cervical spinal disorders concluded that clinical follow-up but not routine cervical radiographs are recommended at least until skeletal maturity is attained in children with cervical spine disorders that do not have current instability [19]. Given these considerations, regular monitoring of the neurological status of patients with this congenital condition may be recommended because anteriorly dislocated occipital bone to atlas results in basilar invagination due to shortening and severe local kyphosis of the craniovertebral junction, which causes neurological impairment, as seen in this present case.

A bifurcated internal occipital crest, which is reported in approximately 3% of the normal population, was identified in this case [20]. Therefore, it is not a rare condition; however, no reports have detailed the process of occipito-cervical fusion in patients with this condition. The insertion of occipital screws into the internal occipital crest was crucial for securing occipital plate fixation; therefore, we separately placed the occipital plates bilaterally and successfully obtained solid bone fusion. Although it remains to be clarified whether bifurcated internal occipital crest can be accompanied by congenital atlanto-occipital dislocation, surgeons should be preoperatively aware of patient-specific anatomy, especially in patients with congenital anomalies.

In conclusion, we have reported the first case of Down syndrome complicated by congenital atlanto-occipital dislocation that demonstrated progressive myelopathy and was successfully treated surgically. Although this condition seems to be extremely rare, we provided detailed information about the clinical course, including surgical treatment for future cases with congenital atlanto-occipital dislocation. The accumulation of similar cases is essential to determine the prognosis or optimal treatment for this congenital condition.
